# Bones in human CYP26B1 deficiency and rats with hypervitaminosis A phenocopy *Vegfa* overexpression

**DOI:** 10.1016/j.bonr.2018.06.006

**Published:** 2018-06-21

**Authors:** Thomas Lind, Roberta Lugano, Ann-Marie Gustafson, Maria Norgård, Arie van Haeringen, Anna Dimberg, Håkan Melhus, Stephen P. Robertson, Göran Andersson

**Affiliations:** a Department of Medical Sciences, Section of Clinical Pharmacogenomics and Osteoporosis, Uppsala University, University Hospital, SE-75185 Uppsala, Sweden; b Department of Immunology, Genetics and Pathology, Science for Life Laboratory, The Rudbeck Laboratory, Uppsala University, SE-75185 Uppsala, Sweden; c Division of Pathology, Department of Laboratory Medicine, Karolinska Institutet, Karolinska University Hospital, SE-14152 Huddinge, Sweden; d Department of Human and Clinical Genetics, Leiden University Medical Center, 2333 ZA Leiden, the Netherlands; e Department of Women's and Children's Health, Dunedin School of Medicine University of Otago, 9054 Dunedin, New Zealand

**Keywords:** CYP26B1, Vitamin A, VEGFA, Microhemorrhage, Human, Rat, Bone

## Abstract

Angulated femurs are present prenatally both in CYP26B1 deficient humans with a reduced capacity to degrade retinoic acid (RA, the active metabolite of vitamin A), and mice overexpressing vascular endothelial growth factor a (Vegfa). Since excessive ingestion of vitamin A is known to induce spontaneous fractures and as the Vegfa-induced femur angulation in mice appears to be caused by intrauterine fractures, we analyzed bones from a *CYP26B1* deficient human and rats with hypervitaminosis A to further explore Vegfa as a mechanistic link for the effect of vitamin A on bone. We show that bone from a human with *CYP26B1* mutations displayed periosteal osteoclasts in piles within deep resorption pits, a pathognomonic sign of hypervitaminosis A. Analysis of the human angulated fetal femur revealed excessive bone formation in the marrow cavity and abundant blood vessels. Normal human endothelial cells showed disturbed cell-cell junctions and increased *CYP26B1* and *VEGFA* expression upon RA exposure. Studies in rats showed increased plasma and tissue Vegfa concentrations and signs of bone marrow microhemorrhage on the first day of excess dietary vitamin A intake. Subsequently hypervitaminosis A rats displayed excess bone formation, fibrosis and an increased number of megakaryocytes in the bone marrow, which are known characteristics of *Vegfa* overexpression. This study supports the notion that the skeletal phenotype in CYP26B1 deficient human bone is caused by excess RA. Our findings suggest that an initial part of the vitamin A mechanism causing bone alterations is mediated by excess Vegfa and disturbed bone marrow microvessel integrity.

## Highlights

• Human CYP26B1 deficit and rat hypervitaminosis A phenocopy Vegf bone overexpression
• Hypervitaminosis A cause rapid microhemorrhage in rat bone marrow.
• Retinoic acid treatment disrupt cell-cell junction integrity between endothelial cells.
• Hypervitaminosis A have a persistent negative effect on rat bone marrow perfusion.
• Hypervitaminosis A rat bones resemble bones of patients with myelofibrotic disorders.

## 1 Introduction

In humans, biallelic mutations in *CYP26B1*, the gene encoding the major enzyme responsible for degradation of excess intracellular retinoic acid (RA), that lead to a substantial reduction in enzyme activity result in severe skeletal anomalies demonstrating the importance of strict regulation of intracellular RA levels for human bone health (Laue et al., 2011) The major skeletal defects were angulated femora, joint synostosis, advanced bone age and calvarial bone hypoplasia. Unrestricted chondrogenesis and aberrant osteoblast-osteocyte transitioning were proposed as the mechanisms behind the joint synostosis and craniosynostosis (a phenotypic feature associated with a hypomorphic *CYP26B1* mutations), respectively (Laue et al., 2011). However, the mechanism behind the angulated femurs in the *CYP26B1* deficient human remained unexplored.

Vitamin A (retinol) is particularly toxic to bone tissue as it is known to cause spontaneous skeletal fractures in experimental animals when administered in excess (Binkley and Krueger, 2000). No animal species has the capability for *de novo* synthesis of vitamin A and it is thus an essential micronutrient. Ingested vitamin A is fat soluble and quickly absorbed but slowly cleared from the body, thus toxicity can occur either from high-dose exposure during a short time or lower intake over more prolonged periods (Melhus, 2011). In line with this, high intake and elevated serum concentrations of vitamin A in humans have been associated with an increased risk of hip fracture (Melhus et al., 1998; Michaëlsson et al., 2003). We and others have shown that the mechanism leading to vitamin A-induced bone fragility in rats involves opposing effects on bone cells within the periosteal (outside) and endosteal (bone marrow side) surfaces of long bones (Kneissel et al., 2005; Lind et al., 2011; Lind et al., 2012; Lind et al., 2013). Thus, excess vitamin A intake leads to increased bone resorption and reduced bone formation at the periosteal surface leading to narrowing of the bone. Importantly, bone diameter is strongly associated with resistance to fracture (Seeman et al., 2001; Ahlborg et al., 2003). Notably, and in addition to bone thinning, excess vitamin A intake also appears to reduce bone resorption and increase bone formation at the endosteal surface. These seemingly contradictory effects seem to occur by direct effects of vitamin A or its metabolites on osteoblasts at the periosteal site together with, secondary, indirect effects on cells at the endosteal/marrow site (Lind et al., 2011; Lind et al., 2012).

There is an abundance of literature describing the negative effects of excess vitamin A and retinoids on the vasculature ranging from observations *in vitro* to clinically detectable effects. Hemorrhage consistently occurs in rodents with hypervitaminosis A, both during fetal development (Fraser and Travill, 1978; Padmanabhan, 1998) and postnatally (Moore and Wang, 1945; Rodahl, 1950; Nerurkar and Sahasrabudhe, 1956; McCarthy et al., 1989; Teelmann, 1989; Takahashi, 1995), RA increases vascular vessel permeability/leakage (Lombardi et al., 1988; Miyoshi et al., 2010; Wang et al., 2013; Kojima et al., 2017), retinoids prescribed to treat skin conditions commonly cause erythema (Khalil et al., 2017), acute promyelocytic leukemia patients receiving RA therapy may develop an unpredictable but frequent complication called retinoic acid syndrome which manifests with fever, respiratory distress, edema, hypotension, and bleeding (Larson and Tallman, 2003; Patatanian and Thompson, 2008), hypervitaminosis A in children or adults consistently show edema (Persson et al., 1965; Rothman et al., 1995; Beste et al., 2016) and, last but not least, mice lacking *Cyp26b1* show vascular defects and hemorrhaging, as well as excess RA signaling and skeletal malformations (Yashiro et al., 2004; Maclean et al., 2009; Bowles et al., 2014). However, the mechanistic basis underling vitamin A-induced bleeding and vascular leakage is still poorly characterized.

Microarray analysis of bone tissue of rats with hypervitaminosis A show increased expression of vascular endothelial growth factor a (*Vegfa*), a potent vascular permeability factor and a key regulator of vascular homeostasis (Lind et al., 2012; Senger et al., 1983). Vegfa is also a powerful regulator of bone tissue as it has been shown that only a brief induction of Vegfa in normally developed, adult mouse bones causes dramatic pathological changes that resemble the secondary manifestations seen in bones of patients with myelofibrotic disorders (Maes et al., 2010). This is particularly interesting as it has been shown that a mouse model of primary myelofibrosis could be rescued by retinoid-antagonist therapy (Hong et al., 2013). Moreover, overexpression of *Vegfa* in mouse bone tissue during development show a remarkable resemblance in femur angulation to that seen in the human fetuses deficient in CYP26B1 (ref. (Laue et al., 2011; Maes et al., 2010; Sharir et al., 2011)). We hypothesize here that excess vitamin A induces *Vegfa* expression and contributes to the bone phenotypes seen in human CYP26B1 deficiency and rat hypervitaminosis A.

To address this we analyzed human fetal bone samples from an individual homozygous for an allele, predicting a p.Arg363Leu substitution, that dramatically reduces CYP26B1 activity (Laue et al., 2011) as well as normal human endothelial and osteoblasts cells exposed to RA. In addition, we analyze rats with hypervitaminosis A with a focus on the known effects of Vegfa overexpression in bone. To identify both early and delayed effects, we analyzed rat samples from the first and second day after ingestion of excess vitamin A as well as samples one week into hypervitaminosis A.

## 2 Materials and methods

### 2.1 Animals and experimental design

This study was carried out in strict accordance with the recommendations in the Guide for the Care and Use of Laboratory Animals of Sweden. The protocol was approved by the Committee on the Ethics of Animal Experiments of the University of Uppsala (Permit Number: C254/7). Seventy male Sprague-Dawley rats, 5 weeks of age, were obtained from Möllegaards Breeding Centre, Ltd. (Skensved, Denmark). They were acclimatized for one week and kept in groups of three animals and had free access to water. The rats were divided into groups, day 1, day 2 and day 4 with 5 animals in each group (+/− extra vitamin A), and day 7 and day 14 with 10 animals in each group (+/− extra vitamin A). They were fed a standard diet (Lactamin R36, Stockholm, Sweden) containing 12 IU vitamin A/g pellet (“Control”), or a standard diet supplemented with 1700 IU vitamin A/g pellet (“Vitamin A”). The control group is a pair-fed group, *i.e.* the control animals were fed the same amount of chow as that consumed by the vitamin A group to keep food intake and body weight gain of the groups the same. The vitamin A was added to the pellets in the form of retinyl palmitate and retinyl acetate. At the end of the experiment, the rats were killed by exsanguination from the abdominal aorta under Eqvitesin anesthesia (chloral hydrate 182 mg/kg, pentobarbital 41.7 g/kg).

### 2.2 Blood analyses

Blood was collected in heparinized tubes centrifuged and plasma aliquots were stored at −70 °C pending biochemical analyses. Commercially available ELISA kits were utilized for measurement of plasma markers Vegfa, sIcam1/CD54 and Tnf-alpha (R&D Systems), according to the manufacturer's instructions. Data represents n = 4–5/group (day 1 and day 2) and n = 9–10/group (day 7).

### 2.3 Immunohistochemistry and histology

Human tissue was obtained after delivery of a still born infant as described in Laue et al. (2011) (Laue et al., 2011). Consent for pathological analysis of tissue was obtained from the parents according to the statues of the Declaration of Helsinki. Human fetal bone was decalcified and embedded prior to sectioning and immunohistochemical analysis using standard techniques (Laue et al., 2011). The rat bone (humerus) preparation and immunohistochemistry have previously been described in detail (Hollberg et al., 2002). The bones from all animals were sectioned in the same orientation in order to make comparable sections. Immunostaining was achieved by for Vegfa (AF564, R&D Systems); Osteopontin as described before (Lind et al., 2011), PECAM1 (AF3628, R&D Systems), DMP1 (M176) (Takara Bio Inc., Japan), Von Willebrand Factor (A0082, Dako) and Cathepsin K (ref. (Hollberg et al., 2008)). Visualization of the antibodies where achieved by incubation with a secondary biotinylated antibody at a dilution of 1:200 in 10% serum and PBS followed by an avidin–biotin–peroxidase complex incubation using the Vectastain ABC-kit (Vector Laboratories) and the substrate diaminobenzidine tetrahydrochloride (DAB, DAKO). Controls were done by omitting the primary antibody. Trichrome Stain (Masson) kit and Reticulin silver plating kit according Gordon & Sweets were used according manufacturer's instructions (Sigma-Aldrich). To obtain data for periosteal thickness, osteoclast count and reticulin stain we pooled measurements on bone sections from rats killed at day 2, 4 and 7. Reticulin positive area was quantified using ImageJ software and the values correspond to the area for reticulin expressed as arbitrary units (AU) relative to endosteal length.

### 2.4 Histomorphometry

This analysis was conducted by Pharmatest Services Ltd., Turku Finland. Histomorphometric parameters were measured from the diaphysis (cortical bone) of the femur (n = 4/group) following the recommendations by the American Society for Bone and Mineral Research Histomorphometry Nomenclature Committee (Parfitt et al., 1987). The analysis was done using BioQuant Osteo II software version 8.12 (BioQuant Image Analysis Corporation, Nashville, TN). Bones were double-labeled with calcein at day 0 and at day 6 prior to the scheduled terminal necropsy at day 7 to measure dynamic parameters. The bone formation rate/bone surface (BFR/BS; μm^3^/μm^2^/day) was determined at the endosteal surface. We used the center of each label as an estimate to calculate the BFR/BS.

### 2.5 Cell culture

Primary human osteoblasts were isolated from bone obtained from male donors undergoing knee replacement surgery and had no reported bone-related pathologies other than osteoarthritis. The osteoblastic phenotype of cells was verified by use of biochemical markers as previously described (Jonsson et al., 1999). The Uppsala University Hospital ethics committee approved this study (Permit Number: Dnr Ups 03-561) and waived the need for consent from these de-identified donors. The primary human osteoblasts were cultured in alpha-MEM (Sigma-Aldrich) supplemented with 10% heat inactivated fetal bovine serum, 2 mM l-glutamine, 100 mg/ml streptomycin and 100 U/ml penicillin (all Sigma-Aldrich). Human dermal microvascular endothelial cells (HDMEC; 3H Biomedical, Uppsala, Sweden) were cultured on gelatin-coated tissue-culture dishes in complete endothelial cell growth medium (EBM-MV2; PromoCell, Heidelberg, Germany). Cells were seeded on 6-well plates and starved 16 h in endothelial culture medium containing 1% fetal bovine serum followed by addition of 400 nM all-trans-retinoic acid (RA) (Sigma-Aldrich). RA was dissolved in 95% ethanol in a dark room under the flow of nitrogen. The 2 mM stock solution was shielded from light and stored at −70 °C until use. At the end of the experiment total RNA was extracted using either the TRI Reagent (Sigma- Aldrich) for osteoblasts or the RNeasy Plus Mini Kit (Qiagen) for the endothelial cells. HDMEC phenotype was analyzed under 20× objective using a bright field microscope (DM IL LED; Leica) attached to a digital camera (DFC450C; Leica). The apoptosis in HDMEC treated with or without retinoic acid (400 nm) during 48 h was assessed by detection of cleaved caspase-3 activity using a colorimetric assay (Caspase-3 Colorimetric Assay Kit, ab39401, Abcam) as indicated by the manufacturer.

### 2.6 Immunofluorescent staining of endothelial cells

HDMEC were plated on 8-well chamber slides, grown until confluency and starved 16 h in endothelial culture medium containing 1% fetal bovine serum followed by 48 h treatment with 400 nM RA. After treatment, cells were fixed in 4% PFA, permeabilized in 1% BSA/0.1% Triton-X100/PBS and blocked in 3% BSA/0.1% Tween-20/PBS. Cells were probed with VE-Cadherin primary antibody (Abcam, ab33168) diluted in blocking buffer at 4 °C overnight and incubated with appropriate Alexa Fluor-conjugated secondary antibody (Invitrogen) diluted in blocking buffer at room temperature for 2 h. Nuclei and cytoskeleton were stained with Hoechst and Texas Red-conjugated phalloidin respectively (all from Life Technologies). After that, cells were washed three times with PBS, mounted with Fluoromount-G (0100-01, Southern Biotech) and kept in 4 °C for imaging. Cells were analyzed using a Leica SP8 confocal microscope with an oil immersion 63× objective. A representative image of at least 5 individual immunofluorescent staining is shown. VE-cadherin was quantified using ImageJ software and the values correspond to the area positive for VE-cadherin expressed as arbitrary units (AU) relative to the cell number/area.

### 2.7 Quantitative RT-PCR

Four hundred ng of total RNA was transcribed to cDNA using the using the High-Capacity cDNA Reverse Transcription Kit (Applied Biosystems). Quantitative real time PCR was performed using inventoried TaqMan Gene Expression Assays for human *CYP26B1* ENSG00000003137 (Hs01011223_m1) and human *VEGFA* ENSG00000112715 (Hs00900055_m1), according to the manufacturer's protocol, on a TaqMan 7000 apparatus. Cycling protocol: 50 °C for 2 min, followed by 95 °C for 10 min and then 40 cycles of 95 °C 15 s followed by 60 °C for 1 min. For standardization, expression levels were normalized to expression level for human *ACTB* ENSG00000075624 (Hs01060665_g1), derived from dilution standard curves of Ct values for each gene. Each experiment was performed at least three times using triplicates.

### 2.8 Statistical analyses

The two-tailed Student's *t*-test was used. In every case, p < 0.05 was considered statistically significant.

## 3 Results

### 3.1 Fetal bones from a human homozygous for a *CYP26B1* mutation show signs of hypervitaminosis A and excess Vegfa exposure

**Fig. 1:**
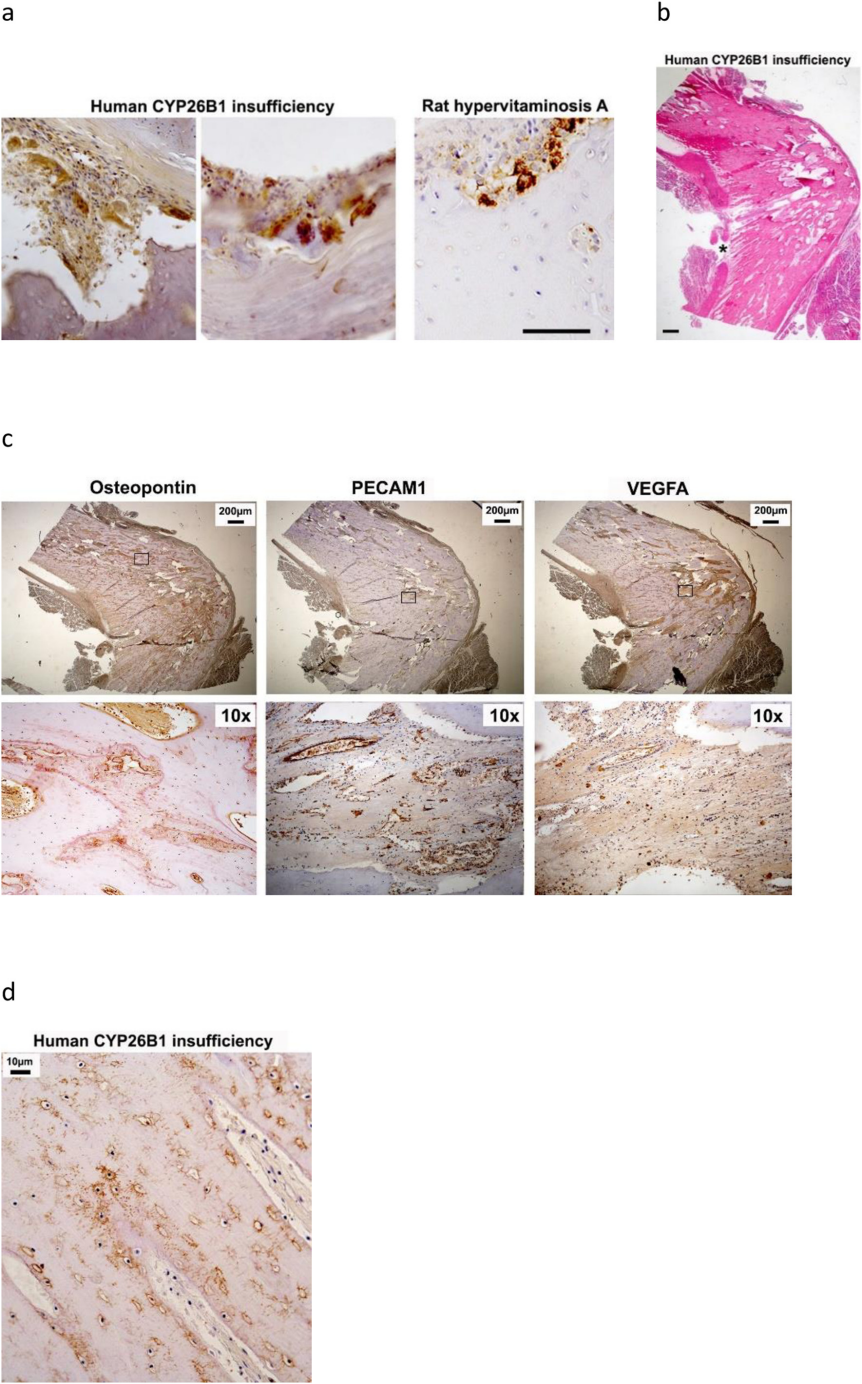
Femur phenotype from an individual with CYP26B1 insufficiency. a) Cathepsin K immunohistochemical staining (brown) of pathological periosteal osteoclast accumulation in sections of fetal bones from an individual with CYP26B1 insufficiency, compared with periosteal osteoclast accumulation from a typical hypervitaminosis A rat bone. Bar = 100 μm. b) Hematoxylin stained section of femur bone tissue from this individual with CYP26B1 insufficiency show angulation at midpoint. Asterisk highlight “striped” bone formation at the angulation point. Bar = 200 μm. c) Osteopontin, PECAM1 and VEGFA immunohistochemical staining (brown) of this human femur bone tissue at the angulation point. Lower panel show pictures at higher magnification from boxed area. d) Dentin matrix protein 1 immunohistochemical staining (brown) of osteocytes and canaliculi in bone tissue at the angulation point of this human fetal femur.

A pathognomonic sign of hypervitaminosis A is piling of many large osteoclasts in deep resorption pits at the periosteal bone surface (Hough et al., 1988). Histological analysis of bones from a previously described individual homozygous for a c.1088G>T mutation (predicting a p.Arg363Leu substitution) in *CYP26B1* showed exactly this pattern (Fig. 1a). In contrast, no osteoclasts were observed in the marrow compartment. Hematoxylin staining of the angulated femur from this individual also demonstrated tissue thickening at the angulation point and also that tissue appeared to grow in “stripes” directed towards the angulation point (Fig. 1b). Osteopontin immunostaining of osteoblasts and cement lines revealed the tissue thickening as excess bone formation which left minimal space for bone marrow at the apex of the angulation (Fig. 1c). In addition, this bone showed abundant immunostaining for PECAM1 and VEGFA (Fig. 1c). Notably, large cavernous capillaries were identified at the endothelial cell layer, whereas smaller bud-like structures were identified using immunohistochemistry for VEFGA. Furthermore, the bone tissue appears mature as indicated by dentin matrix protein 1-positive osteocytes and canaliculi (Fig. 1d).

### 3.2 RA induce *VEGFA* expression and cell-cell junction interruptions in normal human endothelial cell cultures

**Fig. 2:**
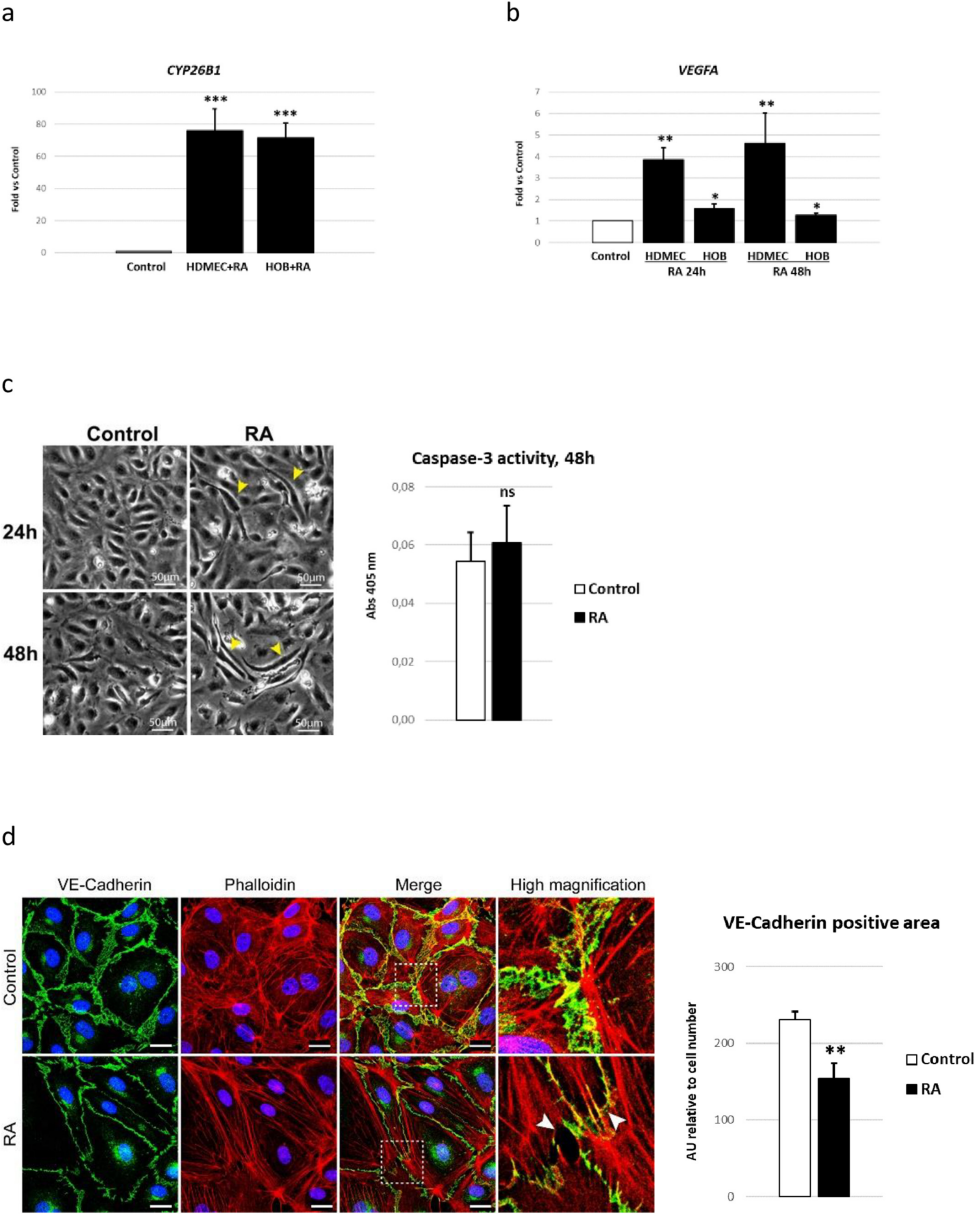
Normal human endothelial cell and osteoblast response to retinoic acid (RA). a) *CYP26B1* mRNA expression in human dermal microvascular endothelial cells (HDMEC) and human primary osteoblasts (HOB) treated with or without 400 nM RA for 24 h (h). b) *VEGFA* mRNA expression in HDMEC and HOB cells treated with or without 400 nM RA for 24 h or 48 h. c) Representative pictures of HDMEC cells treated with or without 400 nM RA for 24 and 48 h. Yellow arrowheads indicate RA induced cell contraction/slimming noticed at both time points. RA did not induce apoptosis as determined by caspase-3 activity. d) Immunofluorescent VE-Cadherin staining of HDMEC cells treated with or without 400 nM RA for 48 h. White arrowheads indicate RA induced gaps in VE-cadherin staining at cell-cell junctions. RA reduced VE-Cadherin staining between endothelial cells. n = 4/treatment. Bar 25 μm. Results are presented as mean ± SD. Student's *t*-test; p < 0.05 *, p < 0.01 ** and p < 0.001 *** compared to control.

Next, we tested if *CYP26B1* expression is associated with RA levels in normal human cells. As shown in Fig. 2a, human dermal microvascular endothelial cells (HDMEC) and primary human osteoblasts (HOB) responded to excess RA by increasing *CYP26B1* mRNA expression approximately 70-fold by 24 h (h). These cells also increased *VEGFA* mRNA expression upon exposure to RA within 24 h, which persisted for at least 48 h (Fig. 2b). The RA treatment further altered cell morphology inducing a slimming of the microvascular endothelial cells, noticed at both 24 h and 48 h after exposure to RA (Fig. 2c). No sign of apoptosis was noted in RA exposed cells (Fig. 2c). In addition, RA treatment disrupted cell-cell junction integrity between endothelial cells, as indicated by discontinuity and reduced staining in VE-Cadherin immunostaining (Fig. 2d).

### 3.3 Early pathological features of hypervitaminosis A in rats

**Fig. 3:**
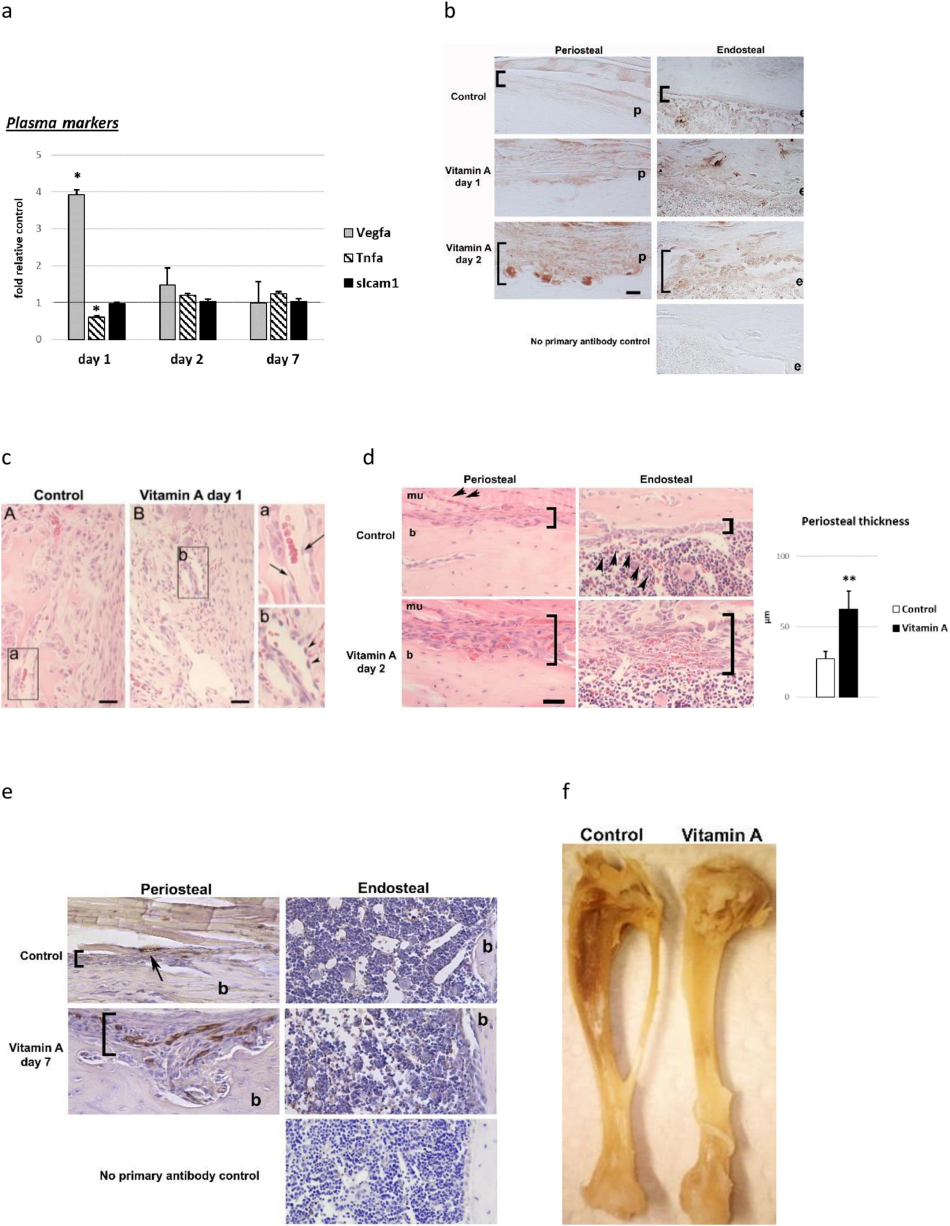
Early pathological features of hypervitaminosis A in young rats. a) Plasma levels of Vegfa, Tnfa and soluble Icam 1 (sIcam1) protein at day 1, 2 and 7 into hypervitaminosis A, determined by ELISA. n = 4–5/group (day 1 and day 2) and n = 9–10/group (day 7). Student's *t*-test; p < 0.05 * compared to control day 1. b) Representative picture of immunohistochemical staining (brown) for Vegfa protein around diaphyseal bone at day 1 and 2 of hypervitaminosis A. Brackets indicate thickness of periosteum and endosteal cell layer. Bar 25 μm. p = periost and e = endost. c) Representative pictures of hematoxylin and eosin (HTX) stained humerus sections from day 1 of hypervitaminosis A. Pictures show tissue infiltration of red blood cells (microbleeding), immediately below the growth plate at day 1 of hypervitaminosis A. Right panel show pictures at higher magnification from boxed area. Arrows indicate endothelial cells lining blood vessels in controls. Arrowheads show contracted endothelial cells lining leaky endothelium in rats with hypervitaminosis A. Bar 25 μm. d) Representative pictures of HTX stained diaphyseal bone from day 2 of hypervitaminosis A and controls. Thickened periosteum show numerous engorged blood vessels whereas the thickened cell layer at the endosteal site show microbleeding. Brackets indicate thickness of periosteum and endosteal cell layer. Arrowheads indicate small intact blood vessel in controls. Bar 25 μm. b = bone and mu = muscle. Data of periosteal thickness is an average of 3 measurements per animal from n = 4 rats/group. Student's *t*-test; p < 0.01 ** compared to control. e) Representative pictures of Pecam1 stained diaphyseal bone from day 7 of hypervitaminosis A and controls. Brackets indicate thickness of periosteal cell layer. Arrow indicate small blood vessel in controls. f) Representative photograph of intact tibia from day 14 of hypervitaminosis A and control. Results are presented as mean ± SD.

**Fig. 4:**
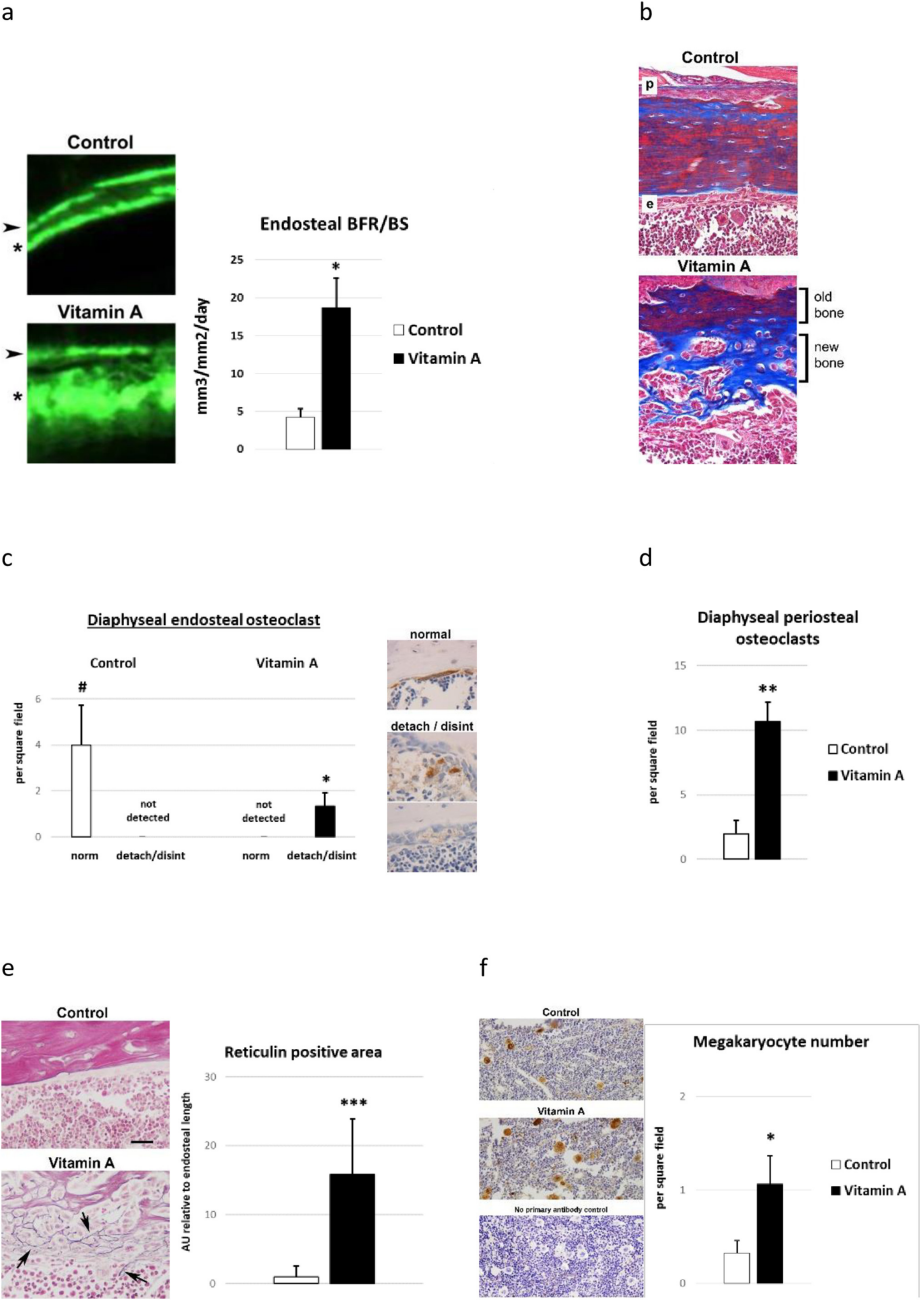
Diaphyseal endosteal bone analysis 7 days into rat hypervitaminosis A. a) Calcein double-labeling of endosteal surface at femur diaphysis. Arrowhead show first label given at day 0 and asterisk indicate second label given 6 days into hypervitaminosis A. Endosteal bone formation rates (BFR/BS) as determined by histomorphometric analysis of calcein double-labeled bones in rats with a high vitamin A intake and pair-fed controls. n = 4 rats/group. b) Masson trichrome staining of diaphyseal bone. In these decalcified bone sections red indicate remodeled compact bone and blue newer (non-remodeled) woven bone. c) Osteoclast number and phenotype at diaphyseal endosteal bone surface. Osteoclast detachment (detach) and osteoclast disintegration (disint). Student's *t*-test; p < 0.05 ^#^ compared to vitamin A normal (norm) and p < 0.05 * compared to control detach/disint. d) Osteoclast number at diaphyseal periosteal bone surface. e) Representative pictures and quantification of stained (black) reticulin fibers (arrows) in the thickened endosteal cell layer in hypervitaminosis A animals. f) Number of von Willebrand factor positive megakaryocytes in diaphyseal bone marrow of hypervitaminosis A and control rats. n = 3 animals/group (2 sections per animal). Results are presented as mean ± SD. Student's *t*-test; p < 0.05 *, p < 0.01 ** and p < 0.001 ***.

To test for early/direct effects on the skeleton we analyzed samples from the first and second day of hypervitaminosis A and making comparisons with paired-fed controls to account for the observation that a high vitamin A intake reduces food intake and consequently growth. Plasma analysis revealed increased concentrations of Vegfa after one day of excess vitamin A ingestion (Fig. 3a). In contrast, plasma levels of tumor necrosis factor alpha (Tnfa), another potent factor involved in bone remodeling and tissue inflammation, was repressed by excess vitamin A ingestion. At day 2 of hypervitaminosis A both Vegfa and Tnfa levels had normalized. Circulating levels of soluble Icam1, also a factor associated with inflammation and endothelial damage, were not altered during the initial 2 days of hypervitaminosis A (Fig. 3a). At day 7 of hypervitaminosis A levels of Vegfa, Tnfa and sIcam1 were normal (Fig. 3a). Immunohistochemical staining of bone tissue showed increased Vegfa protein in both the periosteal and endosteal cell layers in rats that have been ingesting excess vitamin A for only 1 or 2 days (Fig. 3b). Notably, increased numbers of periosteal osteoclasts, a characteristic observation in hypervitaminosis A (see Fig. 1a and Fig. 4d), were clearly apparent after 2 days and stained strongly for Vegfa protein. Additionally, histological analysis at day 1 of hypervitaminosis A, revealed a disrupted microvasculature (microhemorrhage), as represented by scattered red blood cells outside blood vessels, in endosteal tissue close to the active growth plate (Fig. 3c). Notably, cells lining the vessel wall close to vitamin A-induced microbleeding, appeared contracted compared to cells in blood vessels from controls without microbleeding (Fig. 3c). By day 2 of hypervitaminosis A, microbleeding was observed in the thickened cell layer covering the endosteal bone surface in diaphyseal bone (Fig. 3d). In contrast to the endosteal/marrow site in hypervitaminosis A rats, the thickened periosteum displayed increased vascularization, which appeared closer than normal to the periosteal bone surface (Fig. 3d). Further evidence of changes in the vasculature is shown by staining of the endothelium by Pecam1 (day 7) and by pale bones by day 14 (Fig. 3e and f).

### 3.4 Later pathological features of long bones in rats with hypervitaminosis A

After one week of hypervitaminosis A, we observed an increased rate of bone formation at the endosteal surface of diaphyseal bone (Fig. 4a). Importantly, micrographs prepared after calcein double-labeling revealed that the newly-formed endosteal bone was not normal, as the second labeling, administered during high vitamin A intake, was highly diffuse, indicative of non-remodeled woven bone and rapid abnormal mineralization (Fig. 4a). That this labeling represents new bone was further corroborated by Masson trichrome staining (Fig. 4b). Osteoclast counting around diaphyseal bone revealed that excess vitamin A ingestion reduced endosteal osteoclasts involving osteoclast detachment and disintegration (Fig. 4c). In contrast, periosteal osteoclast numbers were higher in hypervitaminosis A animals (Fig. 4d). Additional similarities of bone marrow pathological features observed here in rats with hypervitaminosis A to the known effects of Vegfa overexpression on mouse bones, were excessive reticulin staining, indicative of fibrosis, and an increased number of megakaryocytes (Fig. 4e and f).

## 4 Discussion

Recently it has been shown that mutations in the RA catabolizing enzyme *CYP26B1* lead to effects on long bone development but direct evidence to implicate excessive levels of RA on bone mineralization is incomplete. In this study we have made three important observations by analyzing a femur specimen from a *CYP26B1* deficient human fetus previously described with multiple skeletal anomalies (Laue et al., 2011). First, we show that *CYP26B1* insufficiency induces local pathological accumulation of bone resorbing osteoclasts in the periosteum, similar to that consistently observed in hypervitaminosis A. This observation, together with our recent finding that calvarial hypoplasia previously described in *CYP26B1* insufficiency (Laue et al., 2011) is also characteristic of hypervitaminosis A in mice (Lind et al., 2017), add support to the notion that the skeletal defects observed in the human homozygous for a *CYP26B1* mutation are caused by aberrant RA levels. Secondly, the histological phenotype of the femur angulation caused by *CYP26B1* insufficiency showed striking similarities to both Vegfa overexpressing mouse bones and the healing callus from mechanically fractured mouse bones (Maes et al., 2010; Rot et al., 2014). These similarities, together with the knowledge that the angulation induced by Vegfa overexpression is attributed to a fracture event and not a developmental pattern defect (Sharir et al., 2011), raises the possibility that the angulation in the *CYP26B1* deficient human fetus is caused by an intrauterine fracture. Thirdly, we show that the femur had a minimal marrow cavity at the angulation point, where cortical bone encroached on the marrow cavity. This bone tissue appeared to be mature as it stained for osteopontin at cement lines and for DMP1 in osteocytes and canaliculi. Thus, it appears that *CYP26B1* insufficiency locally induces bone formation in the marrow compartment.

In the subsequent part of this study we analyzed normal human endothelial and osteoblast cells treated with RA and rats with hypervitaminosis A to further corroborate the similarities between *CYP26B1* insufficiency and hypervitaminosis A. These analyses also added evidence that Vegfa and microvascular damage appears as early contributors to the hypervitaminosis A bone pathology. Previously it has been suggested that rapid disorganization of vasculature in embryos induced by RA treatment of pregnant hamsters was responsible for fetal skeletal malformations (Fraser and Travill, 1978). Here in this study, the earliest pathological observation in bones from rats ingesting excessive amounts of vitamin A was bone marrow microhemorrhage at sites of contracted endothelium, which was noticed 1 day into hypervitaminosis A. Along this line, we show that RA induced cell contraction and disrupted cell-cell junction integrity of endothelial cells *in vitro*. In fact, it is known that RA lowers the blood pressure in normal rats and induces hyperpermeability of the capillary endothelium (Lombardi et al., 1988; Miyoshi et al., 2010; Wang et al., 2013; Kojima et al., 2017). In line with this, we show that hypervitaminosis A have a persistent negative effect on bone marrow perfusion. More importantly, human patients treated with RA show an increase in plasma markers of damaged microvascular endothelium within a couple of days of administration (Ryningen et al., 2008).

Additionally, and in agreement with the phenocopy of angulated femurs between *CYP26B1* insufficiency and Vegfa overexpressing mice, we noticed increased levels of Vegfa protein, both in plasma and bone tissue in rats ingesting excessive amounts of vitamin A. Moreover, both human endothelial cells and osteoblasts increased *VEGFA* mRNA expression when exposed to RA. In fact, we previously identified elevated *Vegfa* (and *Cyp26b1*) levels in bone tissue of rats with hypervitaminosis A, using microarray analysis (Lind et al., 2012) and earlier studies show increased production/release of Vegfa from mouse osteoblasts and other cell types/tissues upon RA treatment (Harada et al., 1994; Maeno et al., 2002; Tanabe et al., 2004; Wu et al., 2011; Tan et al., 2015). The rapid normalization in plasma Vegfa after two days of excess vitamin A ingestion, might be explained by the powerful feedback mechanisms (involving Cyp26b1) that exist *in vivo* for reducing harmful effects of excess vitamin A (Lee et al., 2012).

We here also firmly establish that a late effect of hypervitaminosis A in rats is local induction of bone formation in the marrow compartment, which is consistent with the excess bone observed in the marrow compartment of femurs from human *CYP26B1* insufficiency and Vegfa overexpressing mice. Notably, hypervitaminosis A induces bone formation in the marrow compartment despite that RA is a known negative regulator of osteoblast mineralization (Lind et al., 2013; Nallamshetty et al., 2013). Thus, these findings strengthen the hypothesis that RA has indirect effects on endosteal osteoblasts, and adds evidence that this may occur *via* Vegfa, a positive regulator of osteoblast function (Lind et al., 2011; Lind et al., 2012; Lind et al., 2013; Maes et al., 2010; Street and Lenehan, 2009). Excess bone in the marrow compartment in the bones from *CYP26B1* insufficiency may, at least in part, explain the advanced bone age associated with these bones (Laue et al., 2011). These results, together with that the humerus, radius and ulna from the human CYP26B1 insufficient fetus were thinner than normal may indicate that excess vitamin A has deleterious effects on bone strength prenatally. In fact, it was recently shown that high maternal serum retinol levels were negatively associated with offspring neonatal bone diameter at birth (Händel et al., 2016). However, if Vegfa, a well-known potent vascular permeability factor (Senger et al., 1983), is participating as a contributing factor in the cascade of events leading up to the hypervitaminosis A phenotype is not yet proven.

Furthermore, we show here evidence that excess vitamin A have rapid deleterious effects on existing endosteal osteoclasts, which would explain the reduced number of osteoclasts observed at both trabecular and endosteal surfaces in previous studies on rodents exposed to excess retinoid/retinol (Kneissel et al., 2005; Lind et al., 2011). This finding add further evidence of the Vegf involvement in the hypervitaminosis A phenotype as Vegf-overexpressing mice also show reduced number of trabecular osteoclasts (Maes et al., 2010). We also observed an increased number of megakaryocytes and fibrosis in the bone marrow of hypervitaminosis A rats (Maes et al., 2010). Notably, megakaryocytosis and fibrosis and several other pathological bone changes, such as bone thinning and weakening, increased number of periosteal osteoclasts, increased expression of osteoblast marker genes and increased osteoprotegerin expression, that have been described in conjunction with hypervitaminosis A are also features of Vegfa overexpression and myelofibrosis (Kneissel et al., 2005; Lind et al., 2011; Lind et al., 2012; Johansson et al., 2002; Forouhar et al., 1984; Varani et al., 2003; Tefferi, 2005).

Our results indicate that excess Vegfa is associated with the pathological skeletal phenotype in human *CYP26B1* deficiency and in rats with hypervitaminosis A. There is also a strong resemblance between the rat hypervitaminosis A bone phenotype and secondary manifestations seen in bones of patients with myelofibrotic disorders, findings which further support previous studies showing a powerful effect of vitamin A on bone tissue and suggest that an early part of the mechanism is mediated by excess Vegfa and disturbed bone marrow microvessel integrity.

## Ethical approval

Consent for pathological analysis of tissue was obtained from the parents according to the statues of the Declaration of Helsinki. All applicable international, national, and/or institutional guidelines for the care and use of animals were followed. All procedures performed in studies involving animals were in accordance with the ethical standards of the institution or practice at which the studies were conducted.

## Conflict of interests

The authors report no conflicts of interest.

## Transparency document

Transparency document. Image 1

## References

[bb0005] Ahlborg H.G., Johnell O., Turner C.H., Rannevik G., Karlsson M.K. (2003). Bone loss and bone size after menopause. N. Engl. J. Med..

[bb0010] Beste L.A., Moseley R.H., Saint S., Cornia P.B. (2016). CLINICAL PROBLEM-SOLVING. Too much of a good thing. N. Engl. J. Med..

[bb0015] Binkley N., Krueger D. (2000). Hypervitaminosis A and bone. Nutr. Rev..

[bb0020] Bowles J., Secker G., Nguyen C. (2014). Control of retinoid levels by CYP26B1 is important for lymphatic vascular development in the mouse embryo. Dev. Biol..

[bb0025] Forouhar F., Nadel M.S., Gondos B. (1984). Hepatic pathology in vitamin A toxicity. Ann. Clin. Lab. Sci..

[bb0030] Fraser B.A., Travill A.A. (1978). The relation of aberrant vasculogenesis to skeletal malformation in the hamster fetus. Anat Embryol (Berl).

[bb0035] Händel M.N., Moon R.J., Titcombe P. (2016). Maternal serum retinol and β-carotene concentrations and neonatal bone mineralization: results from the Southampton Women's Survey cohort. Am. J. Clin. Nutr..

[bb0040] Harada S., Nagy J.A., Sullivan K.A. (1994). Induction of vascular endothelial growth factor expression by prostaglandin E2 and E1 in osteoblasts. J. Clin. Invest..

[bb0045] Hollberg K., Hultenby K., Hayman A., Cox T., Andersson G. (2002). Osteoclasts from mice deficient in tartrate-resistant acid phosphatase have altered ruffled borders and disturbed intracellular vesicular transport. Exp. Cell Res..

[bb0050] Hollberg K., Marsell R., Norgård M., Larsson T., Jonsson K.B., Andersson G. (2008). Osteoclast polarization is not required for degradation of bone matrix in rachitic FGF23 transgenic mice. Bone.

[bb0055] Hong S.H., Dvorak-Ewell M., Stevens H.Y. (2013). Rescue of a primary myelofibrosis model by retinoid-antagonist therapy. Proc. Natl. Acad. Sci. U. S. A..

[bb0060] Hough S., Avioli L.V., Muir H. (1988). Effects of hypervitaminosis A on the bone and mineral metabolism of the rat. Endocrinology.

[bb0065] Johansson S., Lind P.M., Hakansson H., Oxlund H., Orberg J., Melhus H. (2002). Subclinical hypervitaminosis A causes fragile bones in rats. Bone.

[bb0070] Jonsson K.B., Frost A., Nilsson O., Ljunghall S., Ljunggren O. (1999). Three isolation techniques for primary culture of human osteoblast-like cells: a comparison. Acta Orthop. Scand..

[bb0075] Khalil S., Bardawil T., Stephan C. (2017). Retinoids: a journey from the molecular structures and mechanisms of action to clinical uses in dermatology and adverse effects. J Dermatolog Treat.

[bb0080] Kneissel M., Studer A., Cortesi R., Susa M. (2005). Retinoid-induced bone thinning is caused by subperiosteal osteoclast activity in adult rodents. Bone.

[bb0085] Kojima S., Nishioka C., Chi S., Yokoyama A., Ikezoe T. (2017). In vitro studies on the role of recombinant human soluble thrombomodulin in the context of retinoic acid mediated APL differentiation syndrome. Leuk. Res..

[bb0090] Larson R.S., Tallman M.S. (2003). Retinoic acid syndrome: manifestations, pathogenesis, and treatment. Best Pract. Res. Clin. Haematol..

[bb0095] Laue K., Pogoda H.M., Daniel P.B. (2011). Craniosynostosis and multiple skeletal anomalies in humans and zebrafish result from a defect in the localized degradation of retinoic acid. Am. J. Hum. Genet..

[bb0100] Lee L.M., Leung C.Y., Tang W.W. (2012). A paradoxical teratogenic mechanism for retinoic acid. Proc. Natl. Acad. Sci. U. S. A..

[bb0105] Lind T., Lind P.M., Jacobson A. (2011). High dietary intake of retinol leads to bone marrow hypoxia and diaphyseal endosteal mineralization in rats. Bone.

[bb0110] Lind T., Hu L., Lind P.M., Sugars R. (2012). Microarray profiling of diaphyseal bone of rats suffering from hypervitaminosis A. Calcif. Tissue Int..

[bb0115] Lind T., Sundqvist A., Hu L. (2013). Vitamin a is a negative regulator of osteoblast mineralization. PLoS One.

[bb0120] Lind T., Öhman C., Calounova G. (2017). Excessive dietary intake of vitamin A reduces skull bone thickness in mice. PLoS One.

[bb0125] Lombardi T., Montesano R., Furie M.B., Silverstein S.C., Orci L. (1988). In vitro modulation of endothelial fenestrae: opposing effects of retinoic acid and transforming growth factor beta. J. Cell Sci..

[bb0130] Maclean G., Dollé P., Petkovich M. (2009). Genetic disruption of CYP26B1 severely affects development of neural crest derived head structures, but does not compromise hindbrain patterning. Dev. Dyn..

[bb0135] Maeno T., Tanaka T., Sando Y. (2002). Stimulation of vascular endothelial growth factor gene transcription by all trans retinoic acid through Sp1 and Sp3 sites in human bronchioloalveolar carcinoma cells. Am. J. Respir. Cell Mol. Biol..

[bb0140] Maes C., Goossens S., Bartunkova S. (2010). Increased skeletal VEGF enhances beta-catenin activity and results in excessively ossified bones. EMBO J..

[bb0145] McCarthy D.J., Lindamood C., Gundberg C.M., Hill D.L. (1989). Retinoid-induced hemorrhaging and bone toxicity in rats fed diets deficient in vitamin K. Toxicol. Appl. Pharmacol..

[bb0150] Melhus H., Klemmer P.J. (2011). Vitamin A and bone. Diet, Nutrients, and Bone Health.

[bb0155] Melhus H., Michaëlsson K., Kindmark A. (1998). Excessive dietary intake of vitamin A is associated with reduced bone mineral density and increased risk for hip fracture. Ann. Intern. Med..

[bb0160] Michaëlsson K., Lithell H., Vessby B., Melhus H. (2003). Serum retinol levels and the risk of fracture. N. Engl. J. Med..

[bb0165] Miyoshi T., Arai T., Yamashita K., Sasada M., Uchiyama T. (2010). NB4 cells treated with all-trans retinoic acid generate toxic reactive oxygen species that cause endothelial hyperpermeability. Leuk. Res..

[bb0170] Moore T., Wang Y.L. (1945). Hypervitaminosis A. Biochem. J..

[bb0175] Nallamshetty S., Wang H., Rhee E.J. (2013). Deficiency of retinaldehyde dehydrogenase 1 induces BMP2 and increases bone mass in vivo. PLoS One.

[bb0180] Nerurkar M.K., Sahasrabudhe M.B. (1956). Metabolism of calcium, phosphorus and nitrogen in hypervitaminosis A in young rats. Biochem. J..

[bb0185] Padmanabhan R. (1998). Retinoic acid-induced caudal regression syndrome in the mouse fetus. Reprod. Toxicol..

[bb0190] Parfitt A.M., Drezner M.K., Glorieux F.H. (1987). Bone histomorphometry: standardization of nomenclature, symbols, and units. Report of the ASBMR Histomorphometry Nomenclature Committee. J. Bone Miner. Res..

[bb0195] Patatanian E., Thompson D.F. (2008). Retinoic acid syndrome: a review. J. Clin. Pharm. Ther..

[bb0200] Persson B., Tunell R., Ekengren K. (1965). Chronic vitamin a intoxication during the first half year of life; description of 5 cases. Acta Paediatr. Scand..

[bb0205] Rodahl K. (1950). Hypervitaminosis A in the rat. J. Nutr..

[bb0210] Rot C., Stern T., Blecher R., Friesem B., Zelzer E. (2014). A mechanical Jack-like Mechanism drives spontaneous fracture healing in neonatal mice. Dev. Cell.

[bb0215] Rothman K.J., Moore L.L., Singer M.R., Nguyen U.S., Mannino S., Milunsky A. (1995). Teratogenicity of high vitamin A intake. N. Engl. J. Med..

[bb0220] Ryningen A., Stapnes C., Paulsen K., Lassalle P., Gjertsen B.T., Bruserud O. (2008). In vivo biological effects of ATRA in the treatment of AML. Expert Opin. Investig. Drugs.

[bb0225] Seeman E., Duan Y., Fong C., Edmonds J. (2001). Fracture site-specific deficits in bone size and volumetric density in men with spine or hip fractures. J. Bone Miner. Res..

[bb0230] Senger D.R., Galli S.J., Dvorak A.M., Perruzzi C.A., Harvey V.S., Dvorak H.F. (1983). Tumor cells secrete a vascular permeability factor that promotes accumulation of ascites fluid. Science.

[bb0235] Sharir A., Stern T., Rot C., Shahar R., Zelzer E. (2011). Muscle force regulates bone shaping for optimal load-bearing capacity during embryogenesis. Development.

[bb0240] Street J., Lenehan B. (2009). Vascular endothelial growth factor regulates osteoblast survival - evidence for an autocrine feedback mechanism. J. Orthop. Surg. Res..

[bb0245] Takahashi O. (1995). Haemorrhagic toxicity of a large dose of alpha-, beta-, gamma- and delta-tocopherols, ubiquinone, beta-carotene, retinol acetate and l-ascorbic acid in the rat. Food Chem. Toxicol..

[bb0250] Tan X., Takahashi H., Nishida J., Aoki A., Inoue T., Yanagi Y. (2015). Excessive retinol intake exacerbates choroidal neovascularization through upregulated vascular endothelial growth factor in retinal pigment epithelium in mice. Exp. Eye Res..

[bb0255] Tanabe K., Hirade K., Ishisaki A. (2004). Possible involvement of p44/p42 MAP kinase in retinoic acid-stimulated vascular endothelial growth factor release in aortic smooth muscle cells. Atherosclerosis.

[bb0260] Teelmann K. (1989). Retinoids: toxicology and teratogenicity to date. Pharmacol. Ther..

[bb0265] Tefferi A. (2005). Pathogenesis of myelofibrosis with myeloid metaplasia. J. Clin. Oncol..

[bb0270] Varani J., Fligiel H., Zhang J. (2003). Separation of retinoid-induced epidermal and dermal thickening from skin irritation. Arch. Dermatol. Res..

[bb0275] Wang Y., Han Y., Yang J. (2013). Relaxant effect of all-trans-retinoic acid via NO-sGC-cGMP pathway and calcium-activated potassium channels in rat mesenteric artery. Am. J. Physiol. Heart Circ. Physiol..

[bb0280] Wu J., Hansen J.M., Hao L., Taylor R.N., Sidell N. (2011). Retinoic acid stimulation of VEGF secretion from human endometrial stromal cells is mediated by production of reactive oxygen species. J. Physiol..

[bb0285] Yashiro K., Zhao X., Uehara M. (2004). Regulation of retinoic acid distribution is required for proximodistal patterning and outgrowth of the developing mouse limb. Dev. Cell.

